# Qual o custo hospitalar do desconforto respiratório neonatal para o Sistema Único de Saúde? Análise nacional dos custos diretos, distribuição regional e variação temporal

**DOI:** 10.1590/0102-311XPT220625

**Published:** 2026-10-05

**Authors:** Leticia Araujo Villarinho, Julia Raffin Moura, Mchilanny Bussinguer de Menezes, Emilie Freire Batista, Silvana Alves Pereira, Aline Martins de Toledo

**Affiliations:** 1 Universidade de Brasília, Brasília, Brasil.; 2 Universidade Federal do Rio Grande do Norte, Natal, Brasil.

**Keywords:** Recém-Nascido, Síndrome do Desconforto Respiratório do Recém-Nascido, Custo e Análise de Custo, Unidades de Terapia Intensiva Neonatal, Sistema Único de Saúde, Newborn Infant, Newborn Respiratory Distress Syndrome, Cost and Cost Analysis, Neonatal Intensive Care Units, Unified Health System, Recién Nacido, Síndrome de Dificultad Respiratoria del Recién Nacido, Costos y Análisis de Costo, Unidades de Cuidado Intensivo Neonatal, Sistema Único de Salud

## Abstract

O desconforto respiratório neonatal constitui condição de elevada gravidade clínica e carga econômica para o Sistema Único de Saúde. Este estudo estimou os custos diretos das internações entre 2018 e 2021 e descreveu sua distribuição por diagnóstico, região e período. Realizou-se um estudo de custo de doença, abordagem *top-down*, com autorizações de internação hospitalar de recém-nascidos de 0 a 27 dias. Os valores foram atualizados para dezembro de 2025 e padronizados por mil nascidos vivos. No período, ocorreram 1.449.694 internações neonatais, das quais, 227.130 (15,7%) foram por desconforto respiratório; cerca de metade foi classificada como “não especificada” pela Classificação Internacional de Doenças, 10ª revisão. O gasto total aproximou-se de 1,4 bilhão de Reais, sendo 69,6% referentes ao cuidado em unidades de terapia intensiva neonatal. O custo por mil nascidos vivos variou de R$ 111.625 na Região Norte a R$ 769.847 na Região Sul. A síndrome do desconforto respiratório apresentou a maior mediana de custo por internação (R$ 8.060). Registrou-se redução das admissões por síndrome do desconforto respiratório e aumento por taquipneia transitória, outros desconfortos e diagnósticos não especificados, com queda nas medianas de custo no período. Entre 2020 e 2021, observou-se maior uso de leitos de terapia intensiva neonatal e aumento da mortalidade proporcional nos casos classificados como “não especificados”. A carga econômica é elevada e heterogênea entre as regiões. As estimativas por diagnóstico e região orientam o planejamento da capacidade de terapia intensiva neonatal e políticas de equidade na atenção perinatal.

## Abstract (en)

Neonatal respiratory distress syndrome shows high clinical severity and economic burden for the Brazilian Unified National Health System. This study estimated the direct costs of hospitalizations from 2018 to 2021 and described their distribution by diagnosis, region, and period. A cost-of-disease study with a top-down approach and authorizations for hospitalization of newborns aged from 0 to 27 days was carried out. Values were updated to December 2025 and standardized per 1,000 live births. The period included 1,449,694 neonatal hospitalizations, of which 227,130 (15.7%) were due to respiratory distress (about half of which were classified as “unspecified” by the International Classification of Diseases, 10^th^ revision). Total spending neared BRL 1.4 billion, 69.6% of which referred to care in neonatal intensive care units. The cost per 1,000 live births ranged from BRL 111,625 in the Brazilian North to BRL 769,847 in the South. Respiratory distress syndrome had the highest median cost per hospitalization (BRL 8,060). Admissions decreased due to respiratory distress syndrome but increased due to transient tachypnea, other discomforts, and unspecified diagnoses. Cost medians decreased in the period. A greater use of neonatal intensive care beds and an increase in proportional mortality in “unspecified” cases occurred from 2020 to 2021. Regions show high and heterogeneous economic burden. Estimates by diagnosis and region can guide neonatal intensive care capacity planning and equity policies in perinatal care.

## Resumen

La dificultad respiratoria neonatal es una afección de alta gravedad clínica y carga económica para el Sistema Único de Salud brasileño. Este estudio estimó los costos directos de las hospitalizaciones entre 2018 y 2021 y describió su distribución por diagnóstico, región y período. Se realizó un estudio de costos de enfermedad, con enfoque *top-down*, utilizando autorizaciones de hospitalizaciones de recién nacidos de cero a 27 días. Los valores se actualizaron a diciembre de 2025 y se estandarizaron por cada mil nacidos vivos. Durante el período establecido, se registraron 1.449.694 hospitalizaciones neonatales, de las cuales 227.130 (el 15,7%) se debieron a dificultad respiratoria, aproximadamente la mitad se clasificó como “no especificada” según la Clasificación Internacional de Enfermedades, 10ª revisión. El gasto total se aproximó a los BRL 1.400 millones, de los cuales el 69,6% correspondió a la atención en unidades de cuidados intensivos neonatales. El costo por cada mil nacidos vivos osciló entre BRL 111.625 en la Región Norte y BRL 769.847 en la Región Sur. El síndrome de dificultad respiratoria presentó la mayor mediana de costo por hospitalización (BRL 8.060). Se registró una reducción de las admisiones por síndrome de dificultad respiratoria y un aumento por taquipnea transitoria, otras afecciones y diagnósticos no especificados, junto con una disminución de las medianas de costo durante el período. Entre 2020 y 2021, se observó un mayor uso de camas de cuidado intensivo neonatal y un aumento de la mortalidad proporcional en los casos clasificados como “no especificados”. La carga económica es elevada y heterogénea entre las regiones. Las estimaciones por diagnóstico y región orientan la planificación de la capacidad de cuidado intensivo neonatal y las políticas de equidad en la atención perinatal.

## Introdução

O período neonatal, do nascimento ao 27º dia de vida, corresponde à fase mais vulnerável da infância pela elevada suscetibilidade a agravos clínicos e a rápida evolução de quadros respiratórios e infecciosos ^1^. No Brasil, entre 2018 e 2021, a taxa média de 12 óbitos neonatais por mil nascidos vivos aponta para desafios persistentes na qualidade e no acesso à atenção perinatal ^2^.

Entre as principais causas de morbimortalidade neonatal, destaca-se o desconforto respiratório neonatal, frequentemente associado à prematuridade ^3^ ^,^ ^4^ ^,^ ^5^. A taxa de prematuridade no Brasil esteve em torno de 11% na última década, o que corresponde a cerca de 320 mil nascimentos prematuros por ano ^6^. O desconforto respiratório neonatal constitui uma síndrome clínica heterogênea, marcada por aumento do esforço respiratório, cianose e necessidade variável de suporte ventilatório, com desfechos que alternam entre quadros leves e autolimitados até a insuficiência respiratória grave ^3^ ^,^ ^4^.

Clinicamente, o termo desconforto respiratório neonatal abrange o conjunto de condições incluídas na categoria P22 da Classificação Internacional de Doenças, 10ª revisão (CID-10) e compreende a síndrome do desconforto respiratório (P22.0), taquipneia transitória do recém-nascido (P22.1), outros desconfortos respiratórios (P22.8) e desconforto respiratório não especificado (P22.9) ^7^. Apesar de fisiopatologias distintas, essas condições costumam demandar hospitalizações prolongadas, frequentemente em unidades de terapia intensiva (UTI) neonatal, com custos expressivos para o Sistema Único de Saúde (SUS) ^8^ ^,^ ^9^.

Estudos mostram que complicações respiratórias neonatais figuram entre as principais fontes de gastos hospitalares na primeira infância e, em países de renda média, a internação por síndrome do desconforto respiratório pode custar múltiplas vezes mais do que outras causas neonatais ^9^ ^,^ ^10^. No Brasil, pesquisas indicam que condições respiratórias respondem por parcela substantiva dos custos da prematuridade, sobretudo quando há ventilação mecânica ou uso de surfactante ^11^ ^,^ ^12^.

No país, análises nacionais de custos diretos hospitalares do desconforto respiratório neonatal ainda são escassas. Há, entretanto, literatura sobre desigualdades estruturais (como insuficiência e distribuição desigual de leitos de UTI neonatal e diferenças na cobertura de pré-natal) que podem se refletir em variações regionais de utilização de serviços e de custos em saúde ^13^ ^,^ ^14^ ^,^ ^15^. Além disso, estudos internacionais documentaram alterações em admissões e desfechos neonatais durante a pandemia de COVID-19, e sugerem possíveis efeitos sobre a demanda por cuidado intensivo e sobre mortalidade ^16^ ^,^ ^17^ ^,^ ^18^.

Nesse cenário, compreender a distribuição regional dos custos diretos associados ao desconforyo respiratório neonatal é fundamental para subsidiar políticas voltadas à equidade na saúde neonatal. O Brasil apresenta marcantes desigualdades na disponibilidade de leitos de UTI neonatal e na cobertura de pré-natal, particularmente nas regiões Norte e Nordeste. A criação da Rede Alyne reforça a urgência de reorganizar a atenção perinatal com base em evidências epidemiológicas e econômicas, de modo a aprimorar a eficiência e a equidade na oferta de cuidados especializados ^19^.

Diante desse contexto, o presente estudo teve como objetivo geral estimar os custos diretos hospitalares do desconforto respiratório neonatal no Brasil entre 2018 e 2021 e descrever sua distribuição por subcategoria diagnóstica, região e período.

## Métodos

### Delineamento do estudo

Realizou-se um estudo de custo de doença, sob a perspectiva do SUS, com abordagem *top-down*, baseada na prevalência do desconforto respiratório neonatal no período de 2018 a 2021. O foco foi estimar os custos diretos de internações hospitalares relacionadas ao desconforto respiratório neonatal no setor público de saúde brasileiro ^20^.

### População de estudo e condições de saúde

Foram incluídas Autorizações de Internação Hospitalar (AIH) de recém-nascidos com 0 a 27 dias de vida, com diagnóstico de desconforto respiratório neonatal, conforme a CID-10. Incluímos os códigos: síndrome do desconforto respiratório do recém-nascido (P22.0), taquipneia transitória do recém-nascido (P22.1), outros desconfortos respiratórios do recém-nascido (P22.8) e desconforto respiratório não especificado do recém-nascido (P22.9) ^7^.

### Fonte dos dados

Os dados de internação e custos derivaram do Sistema de Informações Hospitalares do SUS (SIH-SUS), disponibilizado pelo Departamento de Informação e Informática do SUS (DATASUS) e extraídos em 9 de janeiro de 2024. O denominador populacional (nascidos vivos) foi obtido no Sistema de Informações sobre Nascidos Vivos (SINASC). As AIH reúnem informações sobre pacientes, procedimentos, tempo de permanência e custos apurados com base na Tabela Unificada de Procedimentos, Medicamentos e OPM (órteses, próteses e materiais especiais) do SUS (SIGTAP - Sistema de Gerenciamento da Tabela de Procedimentos, Medicamentos e OPM do SUS) ^21^ ^,^ ^22^ ^,^ ^23^.

### Variáveis e processamento

O pré-processamento (filtro e tabulação) foi realizado no TabWin v4.15 (http://www2.datasus.gov.br/DATASUS/index.php?area=060805) e as análises na linguagem R v4.4.0 (http://www.r-project.org). As variáveis contemplaram: região da internação; ano de processamento da AIH; caráter da internação (urgência/eletiva); uso de UTI neonatal (sim/não); diagnóstico principal (CID-10); óbito (sim/não); custo total (R$); custo em UTI neonatal (R$); e idade em dias. Para as comparações populacionais, foram consideradas apenas as declarações de nascidos vivos emitidas em estabelecimentos públicos no período estudado.

### Mensuração e ajuste de custos

O custo total por AIH corresponde à soma de: (a) UTI neonatal; (b) serviços hospitalares (diárias, alimentação, taxas de sala, materiais, medicamentos e exames diagnósticos); e (c) serviços profissionais (honorários médicos e de cirurgiões-dentistas).

Os valores foram corrigidos pela inflação até dezembro de 2024 pelo Índice Nacional de Preços ao Consumidor Amplo (IPCA), emitido pelo Instituto Brasileiro de Geografia e Estatística (IBGE), e descritos no Material Suplementar (https://cadernos.ensp.fiocruz.br/ojs/index.php/csp/article/view/11572/21104) e expressos em Reais (R$) ^24^. Embora o custo de UTI neonatal componha o custo total, procedeu-se a análise específica desse componente para caracterizar a sua participação relativa e o comportamento ao longo do período.

## Análise dos dados

A distribuição das variáveis de custo foi avaliada quanto à normalidade e observada a assimetria, motivo pelo qual optou-se pela apresentação dos dados em mediana e intervalo interquartil (IQR), além das variáveis categóricas por frequências absoluta e relativa. Os resultados foram estratificados por diagnóstico, ano, região, tipo de internação e desfecho (alta/óbito).

Dessa forma, com a exploração das variáveis de custos, nenhum valor discrepante foi excluído da análise principal, os custos extremos foram considerados clinicamente aceitáveis, pois refletem a heterogeneidade clínica real em internações neonatais por desconforto respiratório. Análises de sensibilidade foram conduzidas por meio da exclusão de valores extremos de custo, definidos como observações abaixo do percentil 1 e acima do percentil 99 da distribuição. As estimativas de mediana e IQR foram recalculadas e comparadas às obtidas na amostra completa.

Para as comparações em nível populacional, os custos foram padronizados por mil nascidos vivos, estratificados por condição de saúde e região geográfica do Brasil. A padronização foi realizada dividindo-se os custos pelo número de nascidos vivos ocorridos em estabelecimentos públicos de saúde no mesmo local e período, e multiplicando-se o resultado por mil. A identificação dos estabelecimentos públicos e do número correspondente de nascidos vivos foram obtidos baseando-se nos dados disponibilizados no SINASC, valendo-se do Cadastro Nacional de Estabelecimentos de Saúde (CNES) do local de ocorrência do nascimento. As frequências de nascidos vivos estão descritas no Material Suplementar (https://cadernos.ensp.fiocruz.br/ojs/index.php/csp/article/view/11572/21104).

A variação temporal foi sintetizada por índices anuais, tomando-se 2018 como referência (valor = 1); os anos subsequentes foram expressos relativamente a 2018, estratégia adotada para avaliar a influência do período pandêmico (2020-2021) sobre o número de internações, os óbitos e os custos hospitalares, bem como para descrever as tendências temporais dessas variáveis.

## Aspectos éticos

Foram utilizados os dados secundários, públicos, com acesso aberto e anonimizados, o que dispensa a apreciação por comitê de ética em pesquisa, conforme a *Resolução nº 466*, de 12 de dezembro de 2012 do Conselho Nacional de Saúde ^25^.

## Resultados

### Descrição da amostra

Entre 2018 e 2021, foram registradas 1.449.694 internações neonatais no SUS; 227.130 (15,7%) envolveram desconforto respiratório neonatal. Entre esses registros, 57% foram do sexo masculino e 37% à cor/raça parda. A mortalidade geral foi de 7,3% (16.610 óbitos). As internações de urgência predominaram (97,5%) e 55,2% dos registros foram de admissões em UTI neonatal, contexto em que a mortalidade atingiu 11,2% (Tabela 1).

**Tabela 1:** Distribuição dos registros de hospitalização neonatal por desconforto respiratório segundo características sociodemográficas, diagnósticos, uso de unidade de terapia intensiva (UTI) neonatal, caráter da internação e desfecho. Brasil, 2018-2021.

Características	Centro-oeste	Nordeste	Norte	Sudeste	Sul	Brasil
	n (%)	n (%)	n (%)	n (%)	n (%)	n (%)
	[n = 13.575]	[n = 71.790]	[n = 17.680]	[n = 92.923]	[n = 31.162]	[n = 227.130]
Gênero						
Feminino	5.792 (42,7)	31.960 (44,5)	7.591 (42,9)	39.417 (42,4)	12.935 (41,5)	97.695 (43,0)
Masculino	7.783 (57,3)	39.830 (55,5)	10.089 (57,1)	53.506 (57,6)	18.227 (58,5)	129.435 (57,0)
Raça/Cor						
Amarela	19 (0,1)	1.789 (2,5)	9 (0,1)	337 (0,4)	335 (1,1)	2.489 (1,1)
Branca	2.661 (19,6)	4.341 (6,0)	425 (2,6)	34.154 (36,7)	22.406 (71,9)	64.014 (28,2)
Indígena	205 (1,5)	17 (0,1)	77 (0,5)	9 (0,01)	86 (0,3)	394 (0,2)
Parda	6.309 (46,5)	35.914 (50,0)	10.286 (58,2)	29.530 (31,8)	2.063 (6,6)	84.102 (37,0)
Preta	56 (0,4)	533 (0,7)	259 (1,5)	1.963 (2,1)	906 (2,9)	3.717 (1,6)
Não declarada	4.325 (31,9)	29.196 (40,7)	6.597 (37,3)	26.930 (29,0)	5.366 (17,2)	72.414 (31,9)
Óbito						
Sim	784 (5,8)	5.034 (7,0)	1.582 (8,9)	6.923 (7,5)	2.287 (7,3)	16.610 (7,3)
Não	12.791 (94,2)	66.756 (93,0)	16.098 (91,1)	86.000 (92,5)	28.875 (92,7)	210.520 (92,7)
Caráter de internação						
Eletivo	81 (0,6)	546 (0,8)	563 (3,2)	2.688 (2,9)	1.719 (5,5)	5.597 (2,5)
Urgência	13.494 (99,4)	71.244 (99,2)	17.117 (96,8)	90.235 (97,1)	29.443 (94,5)	221.533 (97,5)
Uso de UTI neonatal						
Sim	5.655 (41,7)	28.988 (40,4)	7.712 (43,6)	60.894 (65,5)	22.103 (70,9)	125.352 (55,2)
Não	7.920 (58,3)	42.802 (59,6)	9.968 (56,4)	32.029 (34,5)	9.059 (29,1)	101.778 (44,8)
Diagnóstico						
P22.0	2.118 (6,0)	10.034 (28,7)	3.418 (9,8)	13.793 (39,4)	5.643 (16,1)	35.006 (15,4)
P22.1	1.399 (5,3)	8.470 (32,4)	2.160 (8,2)	8.287 (31,7)	5.858 (22,4)	26.174 (11,5)
P22.8	3.168 (9,0)	12.695 (23,7)	2.488 (4,6)	27.348 (51,1)	7.825 (14,6)	53.524 (23,6)
P22.9	6.890 (6,1)	40.591 (36,1)	9.614 (8,6)	43.495 (38,7)	11.836 (10,5)	112.426 (49,5)

P22.0: síndrome do desconforto respiratório do recém-nascido; P22.1: taquipneia transitória do recém-nascido; P22.8: outros desconfortos respiratórios do recém-nascido; P22.9: desconforto respiratório não especificado do recém-nascido.

Fonte: Departamento de Informação e Informática do SUS ^21^.

Quanto ao diagnóstico principal (CID-10), o desconforto respiratório neonatal não especificado (P22.9) foi o mais frequente (49,5%), seguido por outros desconfortos respiratórios (P22.8) com 23,6%, síndrome do desconforto respiratório (P22.0) com 15,4% e taquipneia transitória (P22.1) com 11,5%. Dois grupos de procedimentos responderam por 96,4% do total: “Tratamento de transtornos respiratórios e cardiovasculares específicos do período neonatal” (66,3%) e “Tratamento de outros transtornos originados no período perinatal” (30,1%). Regionalmente, o Sudeste concentrou 40,9% dos registros de hospitalização por desconforto respiratório neonatal, e o Centro-oeste apresentou a menor participação (6%). A mortalidade foi mais alta no Norte (8,9%) e baixa no Centro-oeste (5,8%). O uso de UTI neonatal variou amplamente entre as regiões, de 40,4% (Nordeste) a 70,9% (Sul).

### Custos agregados e por componentes

A soma das internações por desconforto respiratório neonatal chega a quase R$ 1,4 bilhão, com 69,6% atribuídos às admissões em UTI neonatal (aproximadamente R$ 1 bilhão), a mediana do custo total foi de R$ 2.768 e a dos custos exclusivos com UTI neonatal foi de R$ 4.651 (os dados de custos estão na Tabela 2). Em análise de sensibilidade, com a exclusão de valores extremos (percentis 1 e 99), as estimativas de mediana e IQR apresentaram variações discretas, sem alteração substantiva dos resultados, sugerindo solidez das estimativas frente à presença de valores extremos. A mediana do custo total reduziu R$ 2 após a exclusão dos valores extremos, e a mediana dos custos com UTI neonatal apresentou redução de R$ 144. Os resultados detalhados da análise de sensibilidade estão apresentados no Material Suplementar (https://cadernos.ensp.fiocruz.br/ojs/index.php/csp/article/view/11572/21104).

**Tabela 2:** Custos das hospitalizações neonatais por desconforto respiratório (valores por 1.000 nascidos vivos), segundo diagnóstico, ano, região, desfecho e caráter da internação. Brasil, 2018-2021.

	Custo total (R$)		Custo UTI neonatal (R$)	
	Soma do custo	Mediana (Q1-Q3)	Soma do custo	Mediana (Q1-Q3)
Diagnósticos *				
P22.0	432.171.490	8.060 (3.223-17.908)	319.865.920	7.630 (3.211-16.991)
P22.1	74.921.047	1.481 (699-3.393)	42.213.729	2.686 (1.387-4.855)
P22.8	320.951.638	2.658 (885-7.020)	224.951.729	4.104 (1.853-9.364)
P22.9	613.374.569	2.353 (831-6.544)	415.820.445	4.162 (1.932-9.017)
Ano				
2018	260.144.352	4.855 (2.081-11.097)	260.144.352	4.855 (2.081-11.097)
2019	250.205.288	4.701 (2.015-10.746)	250.205.288	4.701 (2.015-10.746)
2020	256.698.645	4.507 (1.932-9.658)	256.698.645	4.507 (1.932-9.658)
2021	235.803.538	4.070 (1.744-8.721)	235.803.538	4.070 (1.744-8.721)
Região				
Centro-oeste	70.897.178	1.658 (677-6.871)	46.776.487	5.549 (2.686-10.989)
Nordeste	330.897.720	1.797 (733-5.241)	201.032.089	4.070 (1.932-9.014)
Norte	90.181.263	2.388 (913-6.220)	57.389.436	4.829 (2.081-10.746)
Sudeste	698.614.177	3.413 (1.216-9.012)	508.652.209	4.507 (1.932-10.404)
Sul	250.828.407	4.311 (1.587-10.405)	189.001.603	5.151 (2.211-11.560)
Óbito				
Não	1.327.061.293	2.599 (873-7.590)	921.339.829	4.701 (2.015-10.465)
Sim	114.357.451	4.161 (2.284-7.998)	81.511.994	2.907 (1.387-6.936)
Caráter de internação				
Eletivo	40.138.588	3.103 (1.082-8.357)	29.388.020	4.104 (1.744-10.301)
Urgência	1.401.280.157	2.761 (925-7.612)	973.463.803	4.651 (2.015-10.204)
Total	1.441.418.744	2.768 (929-7.632)	1.002.851.823	4.651 (2.015-10.204)

P22.0: síndrome do desconforto respiratório do recém-nascido; P22.1: taquipneia transitória do recém-nascido; P22.8: outros desconfortos respiratórios do recém-nascido; P22.9: desconforto respiratório não especificado do recém-nascido; Q1: primeiro quartil (percentil 25); Q3: terceiro quartil (percentil 75); UTI: unidade de terapia intensiva.

Fonte: Departamento de Informação e Informática do SUS ^21^.

Nota: os valores monetários foram aproximados e expressos em Reais (R$), atualizados para dezembro de 2025 pelo Índice Nacional de Preços ao Consumidor Amplo/Instituto Brasileiro de Geografia e Estatística.

Entre os diagnósticos, a síndrome do desconforto respiratório (P22.0) apresentou as maiores medianas de custo total (R$ 8.060) e de custo em UTI neonatal (R$ 7.630). O desconforto respiratório neonatal não especificado (P22.9) apresentou a segunda maior mediana de custo com UTI neonatal (R$ 4.162), já outros desconfortos respiratórios (P22.8) apresentaram a segunda maior mediana de custo total (R$ 2.658). Registros cujo desfecho foram óbitos apresentaram mediana de custo total superior àqueles em que o óbito não foi registrado (R$ 4.161 *vs.* R$ 2.599). Entre as regiões, as maiores medianas de custo total ocorreram no Sul (R$ 4.311) e no Sudeste (R$ 3.413). Internações eletivas apresentaram mediana de custo total maior (R$ 3.103) do que as de urgência (R$ 2.761).

Ao padronizar por mil nascidos vivos, o custo nacional de assistência hospitalar ao desconforto respiratório neonatal foi de R$ 292.343. Observou-se uma importante variação regional, com valores que oscilaram de R$ 111.625 na Região Norte a R$ 769.847 na Região Sul, correspondendo a um gradiente de quase seis vezes entre os extremos. Em todas as regiões, o diagnóstico de desconforto respiratório neonatal não especificado (P22.9) concentrou os maiores custos por mil nascidos vivos (Tabela 3).

**Tabela 3:** Custos com assistência hospitalar por 1.000 nascidos vivos, segundo diagnóstico e região, Brasil, 2018-2021.

Diagnóstico	Centro-oeste	Nordeste	Norte	Sudeste	Sul
P22.0	39.126	52.457	40.790	133.834	243.200
P22.1	6.485	10.366	6.477	17.916	62.016
P22.8	31.413	25.792	14.559	118.306	200.748
P22.9	95.961	91.677	49.798	180.179	263.883
Total	172.985	180.291	111.625	450.234	769.847

P22.0: síndrome do desconforto respiratório do recém-nascido; P22.1: taquipneia transitória do recém-nascido; P22.8: outros desconfortos respiratórios do recém-nascido; P22.9: desconforto respiratório não especificado do recém-nascido.

Fonte: Departamento de Informação e Informática do SUS ^21^ ^,^ ^22^.

Nota: valores em Reais (R$), atualizados para dezembro de 2025 (Índice Nacional de Preços ao Consumidor Amplo, Instituto Brasileiro de Geografia e Estatística).

### Variação temporal (2018-2021)

Tendo 2018 como referência (índice = 1), verificou-se uma redução no número de internações por síndrome do desconforto respiratório e aumento nos registros por taquipneia transitória, outros desconfortos respiratórios e desconforto respiratório neonatal não especificado, com destaque para desconforto respiratório neonatal não especificado (+15%) (Figura 1). A mediana do custo por internação apresentou redução em todas as condições, exceto para a taquipneia transitória (3% de aumento no período). A proporção de registros admitidos em UTI neonatal diminuiu para registros de síndrome do desconforto respiratório (-4%) e aumentou para os registros de desconforto respiratório neonatal não especificado (+9%), taquipneia transitória (+7%) e outros desconfortos respiratórios (+7%). Quanto à mortalidade proporcional, verificou-se queda para os registros de síndrome do desconforto respiratório (-12%) e elevação para taquipneia transitória (+6%) e desconforto respiratório neonatal não especificado (+13%); para outros desconfortos respiratórios, houve pico em 2020 (+10%), seguido de retorno a valores semelhantes aos de 2018 em 2021. No conjunto, não emergiram padrões consistentes de aumento ou redução quando se compararam os anos pandêmicos (2020-2021) ao biênio pré-pandemia (2018-2019).

**Figura 1:**
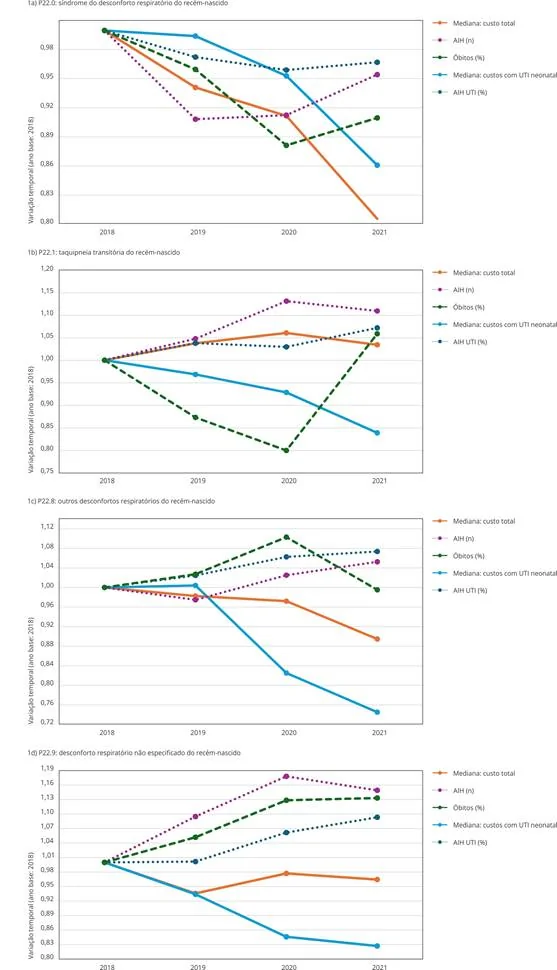
Variação temporal do número de internações, proporção de uso de unidade de terapia intensiva (UTI) neonatal , mortalidade proporcional, custo total (mediana) e custo em UTI neonatal (mediana) por diagnóstico de desconforto respiratório neonatal, Brasil, 2018-2021.

AIH: Autorização de Internação Hospitalar; AIH UTI (%): porcentagem de AIH com o uso de leito de UTI neonatal. Fonte: elaboração própria por meio dos dados coletados para análise.

## Discussão

O presente estudo teve como objetivo estimar os custos diretos hospitalares do no Brasil entre 2018 e 2021, pela análise da distribuição por subcategoria diagnóstica, região e variação temporal. Nossos achados demonstram um custo de aproximadamente R$ 1,4 bilhão para internações neonatais por desconforto respiratório, com a maior parte alocada para internações em UTI neonatal. A mortalidade dos neonatos internados foi de aproximadamente 7,3%. Apesar de o desconforto respiratório neonatal não especificado (P22.9) concentrar a maior frequência e o custo total, a mediana de custo dos registros por síndrome do desconforto respiratório (P22.0) foi aproximadamente 3,4 vezes superior à do desconforto respiratório neonatal não especificado. Registros de UTI neonatal foram a maioria, com destaque para a Região Sul, onde cerca de 71% dos registros foram de UTI neonatal. Nessa mesma região foram estimados os maiores custos por mil nascidos vivos, quase 6 vezes superior ao observado no Norte do país. Observou-se redução das internações por síndrome do desconforto respiratório e aumento nos registros por taquipneia transitória, outros desconfortos respiratórios e desconforto respiratório neonatal não especificado ao longo do período, bem como incremento da proporção de admissões em UTI neonatal nessas últimas condições, em contraste com a redução observada para a síndrome do desconforto respiratório.

Evidências epidemiológicas nacionais demonstram elevada carga de hospitalizações e mortalidade por causas respiratórias no período neonatal, particularmente entre prematuros, que constituem o principal grupo de risco para o desenvolvimento de desconforto respiratório ^26^. A magnitude da carga econômica do desconforto respiratório neonatal para o SUS é igualmente expressiva. Análise recente estimou que cerca de 27% dos custos com internações neonatais no SUS decorreram de condições respiratórias entre 2011 e 2022, totalizando quase R$ 4 bilhões (em valores corrigidos para 2023) ^27^, valor comparável ao custo aproximado de R$ 1,4 bilhão estimado pelo presente estudo em apenas quatro anos.

A integração desses achados indica que as condições respiratórias neonatais não representam apenas um desafio clínico, mas também um dos principais vetores de gasto hospitalar na atenção neonatal. A redução da incidência dessas complicações, especialmente entre prematuros, tem potencial para diminuir a pressão sobre os recursos hospitalares, além de melhorar os desfechos em saúde e reduzir custos indiretos não capturados nas estimativas do presente estudo.

A distribuição de custos e o uso de recursos variaram entre as subcategorias da doença. A síndrome do desconforto respiratório apresentou, de modo consistente, a maior mediana de custo por internação, em linha com a sua fisiopatologia mais grave, a necessidade de suporte ventilatório e o uso de tecnologias de maior custo, como o surfactante ^3^ ^,^ ^5^ ^,^ ^10^ ^,^ ^12^. A taquipneia transitória, condição autolimitada, apresentou custos inferiores, compatíveis com o manejo clínico de menor complexidade ^20^. Por outro lado, o volume de registros foi dominado pelo desconforto respiratório neonatal não especificado, correspondendo à cerca da metade dos registros. A elevada frequência de códigos inespecíficos pode refletir limitações de registro em situações agudas e dificulta a mensuração precisa de incidência e custos por diagnóstico específico ^28^ ^,^ ^29^.

A análise regional revelou iniquidades na alocação de recursos e nos desfechos. Enquanto Sudeste e Nordeste concentraram custos totais elevados pelo maior volume de registros, a Região Sul apresentou a maior mediana de custo por internação e o maior valor ajustado por mil nascidos vivos. Já o Norte, em contexto de maior vulnerabilidade social, exibiu a maior proporção de óbitos e o segundo menor uso de UTI neonatal, refletindo desigualdades estruturais já documentadas como disponibilidade insuficiente de leitos de terapia intensiva e barreiras de acesso geográfico ^13^ ^,^ ^14^ ^,^ ^15^. Disparidades socioeconômicas reforçam a necessidade de políticas de alocação equitativa de recursos, como as propostas pela Rede Alyne, para fortalecer a assistência neonatal nas regiões mais vulneráveis ^19^.

No eixo temporal, observamos redução das admissões por síndrome do desconforto respiratório e aumento dos registros por taquipneia transitória, outros desconfortos respiratórios e desconforto respiratório neonatal não especificado. Em paralelo, verificamos uma queda das medianas de custos. Esse padrão pode decorrer da interação entre os fenômenos: (a) defasagem da Tabela SUS (SIGTAP), com valores de reembolso não atualizados ao ritmo inflacionário, o que reduz o custo ao longo do tempo ^30^; (b) mudança do perfil clínico, com maior proporção de casos menos graves e menor dependência de ventilação ^5^ ^,^ ^31^; e (c) disseminação de práticas custo-efetivas e de tecnologias de suporte mais eficientes (por exemplo, estratégias ventilatórias não invasivas e escolhas de suporte respiratório com melhor relação custo-efetividade) ^32^ ^,^ ^33^. Em particular, a redução de custos associados à síndrome de desconforto respiratório é consistente com a ampliação de boas práticas perinatais (como o uso apropriado de corticosteroide antenatal), associadas à menor necessidade de suporte intensivo ^34^.

As tendências temporais, no geral, não apresentaram padrão claro de alteração, porém, no período pandêmico (2020-2021), identificamos maior uso de UTI neonatal e aumento da mortalidade proporcional nos diagnósticos não especificados. Esses dois fatores podem ter sido influenciados pelas dificuldades administrativas e assistenciais enfrentadas no período da pandemia por COVID-19. Evidências internacionais apontam que a pandemia impactou admissões e resultados neonatais por múltiplos mecanismos, como sobrecarga do sistema (pelo aumento da demanda de casos graves e pelas dificuldades logísticas de fluxo e isolamento) e dificuldades diagnósticas (pela escassez de profissionais ou priorização de recursos), o que pode ter contribuído para a classificação de mais óbitos em categorias inespecíficas ^16^ ^,^ ^17^ ^,^ ^18^.

### Limitações e potencialidades

Limitações inerentes ao uso de dados secundários do DATASUS devem ser reconhecidas. Os custos analisados derivam da tabela SIGTAP, sujeita a desatualizações e pode subestimar os valores reais do cuidado ^30^. A alta frequência de diagnósticos inespecíficos, como o desconforto respiratório neonatal não especificado, limita a precisão da estratificação de custos por patologia e a avaliação da gravidade ^28^ ^,^ ^29^. Adicionalmente, o uso de AIH como unidade de análise e a anonimização dos dados impedem o acompanhamento longitudinal do paciente e inviabilizam análises de reinternação ou seguimento individual. Ainda, nas estimativas de custo por mil nascidos vivos, o denominador refletiu apenas nascidos vivos em estabelecimentos públicos e excluiu aqueles nascidos em leitos cedidos ao SUS em instituições privadas, podendo superestimar taxas regionais conforme a estrutura de oferta privada.

Apesar dessas limitações, as potencialidades do estudo são evidentes em sua abrangência. Embora pesquisas nacionais recentes tenham caracterizado a carga de hospitalizações e mortalidade por causas respiratórias em populações neonatais específicas, como prematuros ^26^, análises abrangentes dos custos diretos hospitalares de diagnósticos específicos do desconforto respiratório neonatal estratificados ainda são escassas. Nesse sentido, o presente estudo avança ao incorporar a dimensão econômica à análise epidemiológica dessas condições. As evidências de custo e a distribuição regional fornecem subsídios concretos para a gestão do SUS. Conclui-se que a carga econômica do desconforto respiratório neonatal é elevada e heterogênea entre as regiões. As estimativas por diagnóstico e região oferecem subsídios para o planejamento da capacidade de terapia intensiva neonatal, a urgência em revisar o financiamento da tabela SIGTAP e para as políticas de equidade na atenção perinatal, fortalecendo a capacidade do SUS em responder às desigualdades.

## Conclusão

O desconforto respiratório neonatal constitui um importante determinante da carga assistencial e econômica da atenção neonatal no SUS, acarretando custos diretos de aproximadamente R$ 1,4 bilhão ao longo de quatro anos, com implicações para a organização dos serviços e a alocação de recursos. Os achados evidenciam desigualdades regionais e limitações na qualidade da informação, indicando oportunidades para o aprimoramento da vigilância e da assistência perinatal. Intervenções voltadas à prevenção da prematuridade e ao manejo qualificado das complicações respiratórias podem gerar ganhos relevantes em eficiência e equidade no sistema de saúde.

## Data Availability

As fontes de informação utilizadas no estudo estão indicadas no corpo do artigo.

## Material Suplementar

Material Suplementar
