# Supplementary material for: What is the cost of neonatal respiratory distress for the Brazilian Unified National Health System? A national analysis of direct costs, regional distribution, and temporal variation
Source: Cad Saude Publica. 2026 Oct 5;42:e00220625. [Article in Portuguese] doi: 10.1590/0102-311XPT220625 (PMC13640035; doi:10.1590/0102-311XPT220625)
Supplement: Supplementary file 1 [file 1678-4464-csp-42-PT220625-s.pdf]

Material Suplementar

Correção Monetária dos Custos pelo IPCA..1
Tabela Suplementar 1: Fatores de correção (IPCA) utilizados...1
Padronização dos custos por 1.000 nascidos vivos..2
Tabela Suplementar 2. Número de nascidos vivos em estabelecimentos públicos no Brasil, por região, entre 2018 e 2021:...3
Análise de sensibilidade..3
Tabela Suplementar 3: Análises de sensibilidade do custo hospitalar para atendimentos ao desconforto respiratório neonatal para o cenário de exclusão de valores extremos (percentis 1 e 99)...4

Correção Monetária dos Custos pelo IPCA

Os valores monetários foram corrigidos utilizando o Índice Nacional de Preços ao Consumidor Amplo (IPCA), calculado pelo Instituto Brasileiro de Geografia e Estatística (IBGE) e operacionalizado pela Calculadora do Cidadão do Banco Central do Brasil (BCB - Calculadora do cidadão). A atualização monetária foi realizada para dezembro de 2025.

A correção foi realizada por meio de fatores multiplicativos específicos para dezembro de cada ano (Tabela Suplementar 1) quando comparados a dezembro de 2025. A fórmula seguinte expressa o método de cálculo utilizado:

$$\text{Custo corrigido} = \text{Custo original} \times \text{Fator de correção}$$

Tabela S1 Fatores de correção (IPCA) utilizados**.**

Ano	Fator de correção
2018	1.44884310
2019	1.40290030
2020	1.34491960
2021	1.21450060
2022	1.14683190
2023	1.09552270
2024	1.04461840
2025	1.00000000

A correção foi aplicada às variáveis de custo das Autorizações de Internações Hospitalares do SIH-SUS: VAL_TOT e VAL_UTI, utilizando o software R.

Código utilizado (Linguagem R):

```
fatores_correcao <- c("2018" = 1.44884310,
                "2019" = 1.40290030,
                "2020" = 1.34491960,
                "2021" = 1.21450060,
                "2022" = 1.14683190,
                "2023" = 1.09552270,
                "2024" = 1.04461840,
                "2025" = 1)
 df <- df %>%
   mutate(
       val_tot_corrigido = VAL_TOT *
fatores_correcao[as.character(ANO_CMPT)],
       val_uti_corrigido = VAL_UTI *
fatores_correcao[as.character(ANO_CMPT)],
```

Todas as análises do estudo foram feitas com base nas variáveis calculadas: val_tot_corrigido e val_uti_corrigido. Todos os valores foram expressos em Reais (R$).

Padronização dos custos por 1.000 nascidos vivos

Para comparações populacionais, os custos foram padronizados por 1.000 nascidos vivos (NV) em análises de custo por condição de saúde e por região do Brasil. A padronização foi realizada dividindo-se os custos totais pelo número de NV em estabelecimentos públicos de saúde no mesmo local e período, multiplicando-se o resultado por 1.000.

Fórmula utilizada:

$$\text{Custo por mil NV} = \frac{\text{Custo total corrigido}}{\text{N° de NV}} \times 1.000$$

O número de NV foi obtido a partir do Sistema de Informações sobre Nascidos Vivos (SINASC), considerando apenas nascimentos ocorridos em estabelecimentos públicos identificados via CNES/TabWin.

Tabela S2 Número de nascidos vivos em estabelecimentos públicos no Brasil, por região, entre 2018 e 2021.

Região	Ano				Total
	2018	2019	2020	2021	
Centro-oeste	107.820	106.283	98.166	97.576	**409.845**
Nordeste	477.065	463.086	445.836	449.363	**1.835.350**
Norte	208.800	207.555	192.802	198.739	**807.896**
Sudeste	414.341	402.125	377.374	357.828	**1.551.668**
Sul	82.735	82.169	81.150	79.762	**325.816**

Análise de sensibilidade

A análise de sensibilidade excluiu valores extremos (percentis 1 e 99) de custo total e custo com UTI neonatal. A exclusão foi feita separadamente para os custos totais na amostra completa e para os custos de UTI neonatal no subgrupo de pacientes com uso de UTI, resultando na exclusão de 2% da amostra completa (custo total) e 1% da amostra de admissões em UTI neonatal.

As variações dos custos estão descritas na Tabela Suplementar 3. As estimativas de mediana apresentaram variações R$2 para custo total, enquanto a mediana dos custos com UTI neonatal apresentou redução de R$ 144.

Tabela S3 Análises de sensibilidade do custo hospitalar para atendimentos ao desconforto respiratório neonatal para o cenário de exclusão de valores extremos (percentis 1 e 99).

Parâmetro	Custo total	Mediana (Q1 - Q3)	Custo com UTI neonatal	Mediana (Q1 - Q3)
Amostra completa	1.441.418.744	2.768 (929-7.632)	1.002.851.823	4.651 (2.015-10.204)
Amostra em extremos	1.311.996.828	2.766 (951-7.459)	930.487.108	4.507 (1.932-9.884)

Nota: os custos estão expressos em Reais (R$) e corrigidos pela inflação conforme o Índice Nacional de Preços ao Consumidor Amplo (IPCA) para dezembro de 2025.

Código utilizado (Linguagem R):

```r
### Análise de sensibilidade: excluindo valores extremos (p1–p99)



# Identificando limites

lim_tot <- quantile(df$val_tot_corrigido, c(0.01, 0.99), na.rm = TRUE)

      # UTI (somente quem usou UTI)

      lim_uti <- df %>%

        filter(USO_UTI == "Sim") %>%

        summarise(

          p1 = quantile(val_uti_corrigido, 0.01, na.rm = TRUE),

          p99 = quantile(val_uti_corrigido, 0.99, na.rm = TRUE)

        )



#### Base trimmed

# Trim para custo total

SIH_tot_trim <- df %>%

  filter(val_tot_corrigido >= lim_tot[1],

         val_tot_corrigido <= lim_tot[2])
```

```
# Trim para custo UTI (apenas subgrupo)

SIH_uti_trim <- df %>%

  filter(USO_UTI == "Sim") %>%

  filter(val_uti_corrigido >= lim_uti$p1,

         val_uti_corrigido <= lim_uti$p99)



# análise:



sensibilidade <- bind_rows(

    tibble(

    base = "Original",

    soma_tot = sum(df$val_tot_corrigido),

    mediana_tot = median(df$val_tot_corrigido, na.rm = TRUE),

    p25_tot = quantile(df$val_tot_corrigido, 0.25, na.rm = TRUE),

    p75_tot = quantile(df$val_tot_corrigido, 0.75, na.rm = TRUE),



    mediana_uti = df %>%

      filter(USO_UTI == "Sim") %>%

      summarise(m = median(val_uti_corrigido, na.rm = TRUE)) %>%
```

```
    pull(m),

  p25_uti = df %>%
    filter(USO_UTI == "Sim") %>%
    summarise(q = quantile(val_uti_corrigido, 0.25, na.rm = TRUE)) %>%
    pull(q),

  p75_uti = df %>%
    filter(USO_UTI == "Sim") %>%
    summarise(q = quantile(val_uti_corrigido, 0.75, na.rm = TRUE)) %>%
    pull(q)
),

tibble(
  base = "Sem extremos (p1–p99)",
  soma_tot = sum(SIH_tot_trim$val_tot_corrigido),
  mediana_tot = median(SIH_tot_trim$val_tot_corrigido, na.rm = TRUE),
  p25_tot = quantile(SIH_tot_trim$val_tot_corrigido, 0.25, na.rm = TRUE),
  p75_tot = quantile(SIH_tot_trim$val_tot_corrigido, 0.75, na.rm = TRUE),
```

```
    som_uti = sum(SIH_uti_trim$val_uti_corrigido),

    mediana_uti = median(SIH_uti_trim$val_uti_corrigido, na.rm = TRUE),

    p25_uti = quantile(SIH_uti_trim$val_uti_corrigido, 0.25, na.rm = TRUE),

    p75_uti = quantile(SIH_uti_trim$val_uti_corrigido, 0.75, na.rm = TRUE)

  )

)



# Número de registros excluídos:

        ##TOT

perc_tot <- (nrow(df) - nrow(SIH_tot_trim)) / nrow(df) * 100

        ## UTI

orig_uti <- df %>% filter(USO_UTI == "Sim")

perc_uti <- (nrow(orig_uti) - nrow(SIH_uti_trim)) / nrow(orig_uti) * 100
```
